# Recent Advances in Dual Disorders (Addiction and Other Mental Disorders)

**DOI:** 10.3390/jcm12093315

**Published:** 2023-05-06

**Authors:** Marta Torrens, Ana Adan

**Affiliations:** 1 Addiction Research Group (GRAd), Neuroscience Research Program, Hospital del Mar Medical Research Institute (IMIM), 08003 Barcelona, Spain; mtorrens@psmar.cat; 2 School of Medicine, Universitat de Vic-Central de Catalunya, 08500 Vic, Spain; 3 Psychiatry Department, School of Medicine, Universitat Autònoma de Barcelona (UAB), 08093 Cerdanyola del Vallès, Spain; 4 Department of Clinical Psychology and Psychobiology, School of Psychology, University of Barcelona, Passeig de la Vall d’Hebrón 171, 08035 Barcelona, Spain; 5 Institute of Neurosciences, University of Barcelona, 08035 Barcelona, Spain

In clinical mental health practice, the presence of Dual Disorders (DDs), defined as the comorbidity of at least one Substance Use Disorder (SUD) and another mental disorder in the same person [1,2], is very high and in recent years, the epidemiological data have been steadily increasing. It can be affirmed that the existence of DDs is more the rule than the exception in the care setting. Currently, a multidisciplinary and comprehensive response to the needs of persons with DDs is required. Our previous publication of a first Special Issue on the subject, prepared before the COVID-19 pandemic, compiled a set of topics, from basic to clinical perspectives, with a very positive impact on scientists and professionals in the field [3]. The evidence leaves no doubt about the need for and interest in considering the existence of psychiatric comorbidities in the therapeutic management of patients with SUD. Additionally, it is even more important, if possible, to carry it out comprehensively by expert interdisciplinary teams since dual patients have greater difficulties in both clinical management and stabilization compared to those with only SUD or other mental health disorders (i.e., increases admissions in emergency rooms, hospitalizations, suicide, etc.).

There is currently a long way to go in understanding DDs and with this second Special Issue, we aimed to compile recent advances, considering different levels of approach that provide interesting data, with a view to being transferred as soon as possible to health care. In addition, the impact of the COVID-19 pandemic has already had a negative impact on mental health, especially in the young population. Thus, the detection of both substances and behavioral addictions and many other mental disorders (major depression, anxiety, eating disorders, etc.) has been alerted. All of this takes place in health systems that are already traditionally stressed in the field of mental health, regardless of the country and the model of care, and where there is a need for specialized centers and professionals trained in the management of DDs. This situation will only be overcome with rigorous work from multiple approaches (biological, psychological, social) that allows for inter-disciplinary integration which will result in future advances in knowledge and overcoming the deficits that exist today and new opportunities to improve them.

Puértolas-Gracia et al. [4] showed in a cohort study of 1356 patients with alcohol or cocaine use disorder, which were admitted to treatment in the public addiction outpatient services in Barcelona, that the lifetime prevalence of screening positive for DDs was 74%, with depression being the most frequent (76.4%). These patients were more frequently women, younger, unemployed, reported higher polysubstance use, poorer self-perceived health, and other medical conditions. The results highlighted the prevalence and clinical relevance of DDs and the need to screen to diagnose and treat properly in these services for addiction disease. In the study of Horigian et al. [5], they confirmed the existence of accidental overdoses and intentional overdoses, and even suicidal attempt, in patients with SUD. In a novel way, they use the Concise Health Risk Tracking Self-Report, a measure that assesses associated symptoms related to suicide as ideation, intent, pessimism, lack of social support, helplessness, and despair. Both studies [4,5] strongly support that entry into treatment for SUD as an invaluable opportunity to assess the presence of other mental disorders or DDs, evaluate suicide risk, and apply adequate treatment and early prevention plans.

However, not only those who request outpatient treatment have a high presence of DDs. Ferrer-Farré et al. [6] studied the existence of DDs among patients with SUD admitted to a general hospital for any pathology and attended by a consultation liaison addiction service, and its association with addiction severity and quality of life. They applied a gender perspective in the study related to the increase in interest in the topic in health in general and in addiction diseases in particular [7]. Again, although the most prevalent DD was depression for both sexes (33.8%), it was higher in women (46.2%) than men (30.9%). Additionally, when DDs were present, women suffered a worse quality of life than men. The study confirms a high prevalence of DDs among patients with SUD admitted to a general hospital for any pathology, and its being associated with a worse quality of life, particularly in women.

Traynor and colleagues [8] developed a trial of patient navigation to reduce barriers to care in people with HIV (human immunodeficiency virus) who reported substance use. Of the 801 participants, 55.3% had a history of substance use treatment in the 6 months prior to treatment, about one-third reported injection drug use in the last 12 months, and 22% had a recorded psychiatric history or DD. Greater severity of alcohol and drug use, less readiness for substance use treatment, and more negative attitudes towards drug treatment were indicators associated with the profiles of patients with increased barriers to care. The results emphasize the need to identify and meet needs at the complex intersection of substance use and HIV services, whose integration can improve patient outcomes.

In recent years, interest in the study of personality traits in DD patients has shown to have differences with respect to those with diagnoses of only SUD or only comorbid mental disorder. It is currently suggested that this could be an endophenotype whose consideration would improve both prevention and treatment programs in mental health [9]. This may be specifically useful in the comorbidity between SUD and personality disorders. The research by De la Rosa-Cáceres et al. [10] focused on the study of the personality facets of the Alternative Model of Personality Disorder of DSM-5 [11] in patients with alcohol use disorder by means of a network analysis. The data show a connection with the facets related to the disinhibition (risk taking, irresponsibility, impulsivity, and rigid perfectionism) and antagonism (callousness, grandiosity, and hostility) domains, which are also associated with premature patient dropout. These facets are relevant to antisocial and borderline personality disorder diagnoses, and their assessment can help to determine whether the personality disorder of patients with SUD is primary or secondary.

Restrictions due to the COVID-19 pandemic had an evident impact on the attention of specialized addiction centers. The study by Mancheo-Velasco et al. [12] evaluated the admissions to treatment of dual patients together with their main sociodemographic and clinical characteristics, considering three key periods in the first wave of the pandemic in Spain: pre-confinement, confinement, and post-confinement. The period of confinement meant there was a large decrease in admissions in general, although in percentage terms, the number of dual patients even increased. There are few differences in the sociodemographic and clinical profile of the patients admitted between time periods, although the increase during the confinement of women, opioid users, and those with mixed anxiety–depressive disorders stand out. Among other indicators, during the confinement, a decrease in the number of toxicological tests and planned therapeutic sessions was observed, even though the patients attended a greater percentage of scheduled sessions.

In recent years, great progress has been made in the development of explanatory models through machine learning in mental health, which could improve both the detection and diagnosis of multiple disorders [13] and be a future tool for precision psychiatry. The research by Oliva et al. [14] is the first to apply machine learning to predict the presence of comorbid SUD in bipolar disorder and using random forest models to identify the type of SUD (any, alcohol, and alcohol with at least another). Although with a low or moderate specificity of the models, due to the consideration of socio-demographical or clinical factors alone, alcohol use disorder with at least one other SUD correctly classifies up to 75% of the sample studied. In addition, this consumption pattern of bipolar patients was positively associated with a hypomanic episode at the onset of bipolar disorder and the presence of hetero-aggressive behavior.

Circadian rhythmicity is an aspect seldom studied in patients with DDs; most of the previous research focuses on sleep problems and/or disorders. The work of Serrano-Serrano et al. [15], studying the circadian rhythm of distal skin temperature and the sleep–wake rhythm, observed that in dual patients with severe mental illness (schizophrenia, bipolar disorder, and major depression) undergoing treatment, after three or more months of abstinence, there is a normalization of sleep, but a more marked impairment of wakefulness persists in dual patients with schizophrenia and bipolar disorder. Consideration of the treatment modality shows a more morning-focused pattern with a better quality of both sleep and wakefulness in residential patients compared to outpatients, regardless of severe mental illness. The data suggest that including in the treatment aspects of regular time habits (activity, intake, exposure to light, etc.) and with a morning-type pattern to promote rhythmic reorganization, this can improve adherence to treatment and the prevention of relapses, especially in outpatients.

The Adan and Navarro [16] protocol proposes a characterization of dual patients, focused on comorbid major depression and schizophrenia, with aspects of genetic polymorphisms, circadian rhythmic functioning, neurocognition, and personality traits with the aim to elucidate the possible markers of vulnerability and prognosis (with a follow-up of one year) useful in clinical practice. In this direction, the same group of researchers has suggested that in dual schizophrenia, the evaluation of the circadian rhythmic expression may be a biomarker [17]. In addition, it is proposed that we carry out a pioneering study with a light exposure intervention in SUD and dual depressed outpatients of both sexes who show difficulties in rhythmic reorganization during treatment.

Finally, the study carried out by Vintró-Alcaraz et al. [18] with gambling disorder outpatients under group cognitive–behavioral treatment is one of the first to explore substance use (tobacco, alcohol, and illegal drugs) in this behavioral addiction. It should be noted that the consumption of substances in these patients exceeds 55%, and that those with the presence of gambling-related illegal acts present a higher likelihood of substance use, specifically tobacco and illegal drugs. These results should encourage future studies on gambling disorders to consider the coexistence of SUD and its implications both in committed gambling related-offenses and in adherence to treatment.
